# Atmospheric Nitrogen Deposition at Two Sites in an Arid Environment of Central Asia

**DOI:** 10.1371/journal.pone.0067018

**Published:** 2013-06-26

**Authors:** Kaihui Li, Xuejun Liu, Wei Song, Yunhua Chang, Yukun Hu, Changyan Tian

**Affiliations:** 1 Key Laboratory of Biogeography and Bioresource in Arid Land, Xinjiang Institute of Ecology and Geography, Chinese Academy of Sciences, Urumqi, China; 2 College of Resources and Environmental Sciences, China Agricultural University, Beijing, China; Glasgow University, United Kingdom

## Abstract

Arid areas play a significant role in the global nitrogen cycle. Dry and wet deposition of inorganic nitrogen (N) species were monitored at one urban (SDS) and one suburban (TFS) site at Urumqi in a semi-arid region of central Asia. Atmospheric concentrations of NH_3_, NO_2_, HNO_3_, particulate ammonium and nitrate (pNH_4_^+^ and pNO_3_^−^) concentrations and NH_4_-N and NO_3_-N concentrations in precipitation showed large monthly variations and averaged 7.1, 26.6, 2.4, 6.6, 2.7 µg N m^−3^ and 1.3, 1.0 mg N L^−1^ at both SDS and TFS. Nitrogen dry deposition fluxes were 40.7 and 36.0 kg N ha^−1^ yr^−1^ while wet deposition of N fluxes were 6.0 and 8.8 kg N ha^−1^ yr^−1^ at SDS and TFS, respectively. Total N deposition averaged 45.8 kg N ha^−1^ yr^−1^at both sites. Our results indicate that N dry deposition has been a major part of total N deposition (83.8% on average) in an arid region of central Asia. Such high N deposition implies heavy environmental pollution and an important nutrient resource in arid regions.

## Introduction

Asia is one of the most important regions of the world in the context of atmospheric aerosol loading because of the presence of growing economies in China, India and other Asian countries [1]. Industrialization, urbanization, economic growth and associated increasing energy demands have resulted in profound deterioration of urban air quality [2], [3]. Anthropogenic activities have greatly increased the emissions of reactive nitrogen (Nr) species [4], with a consequent increase in atmospheric deposition of Nr species. Elevated N deposition induced by human activities will contribute to a number of negative effects such as declining biodiversity, indirect emission of N_2_O, eutrophication of aquatic ecosystems and soil acidification [5]–[7]. Sutton et al. [8] recently estimated the total annual cost of excess Nr in the European Union to be from 70 to 320 billion euros. Excess Nr deposition has therefore become an important public concern due to its close relationship with human health, biodiversity and climate change [9].

Drought is consistently predicted to become particularly severe in arid regions, and these relatively dry regions cover about 30% of the terrestrial biosphere [10] and play a significant role in the global N cycle. Urumqi is the capital city of Xinjiang Uygur Autonomous Region and is located in the center of the arid regions of central Asia to the north of Taklimakan desert and to the south of Gurebantonggute desert. It is one of the most rapidly growing large cities worldwide. Moreover, the urban area of Urumqi is surrounded by the Tianshan Mountains from three directions with peaks up to 5000 m, and there is only one mouth facing north [11]. Together with rapid economic development, Chinese energy consumption has increased by 10% annually since 2000 compared to annual increases of only 4% from 1980 to 2000 [12]. The combustion of fossil fuels by industry and vehicles has produced a significant increase in air pollution in recent years, with rates of consumption of diesel oil, crude oil, raw coal and coke increasing by 12, 27, 68 and 92%, respectively [13]. Furthermore, N fertilizer application has also increased steadily since the 1980s due to the expansion of cotton and cereal/fruit/vegetable production in Xinjiang. NH_3_ emissions from fertilized croplands will be high due to the high N application rates and alkaline soil conditions in this region. We expect increasing atmospheric Nr pollution in this region. On the other hand, N limitation is often a primary or co-occurring factor limiting crop yields in arid regions, but atmospheric Nr deposition can be an important source of nutrients and inputs of Nr deposition have an important fertilizing effect in arid regions [14]. Nr deposition also plays an important role in the nutrient cycle of natural ecosystems or low input agriculture throughout China. For example, Liu et al. [15] estimated total N input from wet and dry deposition and found the values to be 15 Tg N yr^−1^ for the whole country in the 2000s, equal to about 50% of N fertilizer use nationwide. This is an important nutrient input to croplands from the atmosphere. However, very little is known about the influence of air pollutants and the dynamics of Nr dry deposition in arid terrestrial ecosystems. There have been several investigations of seasonal chemical properties of wet deposition [16], but there has been no investigation of N dry deposition in Urumqi because of the complexity of dry deposition monitoring. The current study aimed to answer three basic questions, namely the possibility of differences in ambient concentrations of Nr species between urban and suburban sites in central Asia, atmospheric Nr pollution dynamics and the relative contribution of N dry deposition to total atmospheric (wet plus dry) deposition in this arid environment.

## Materials and Methods

### Monitoring Sites

**Figure 1:**
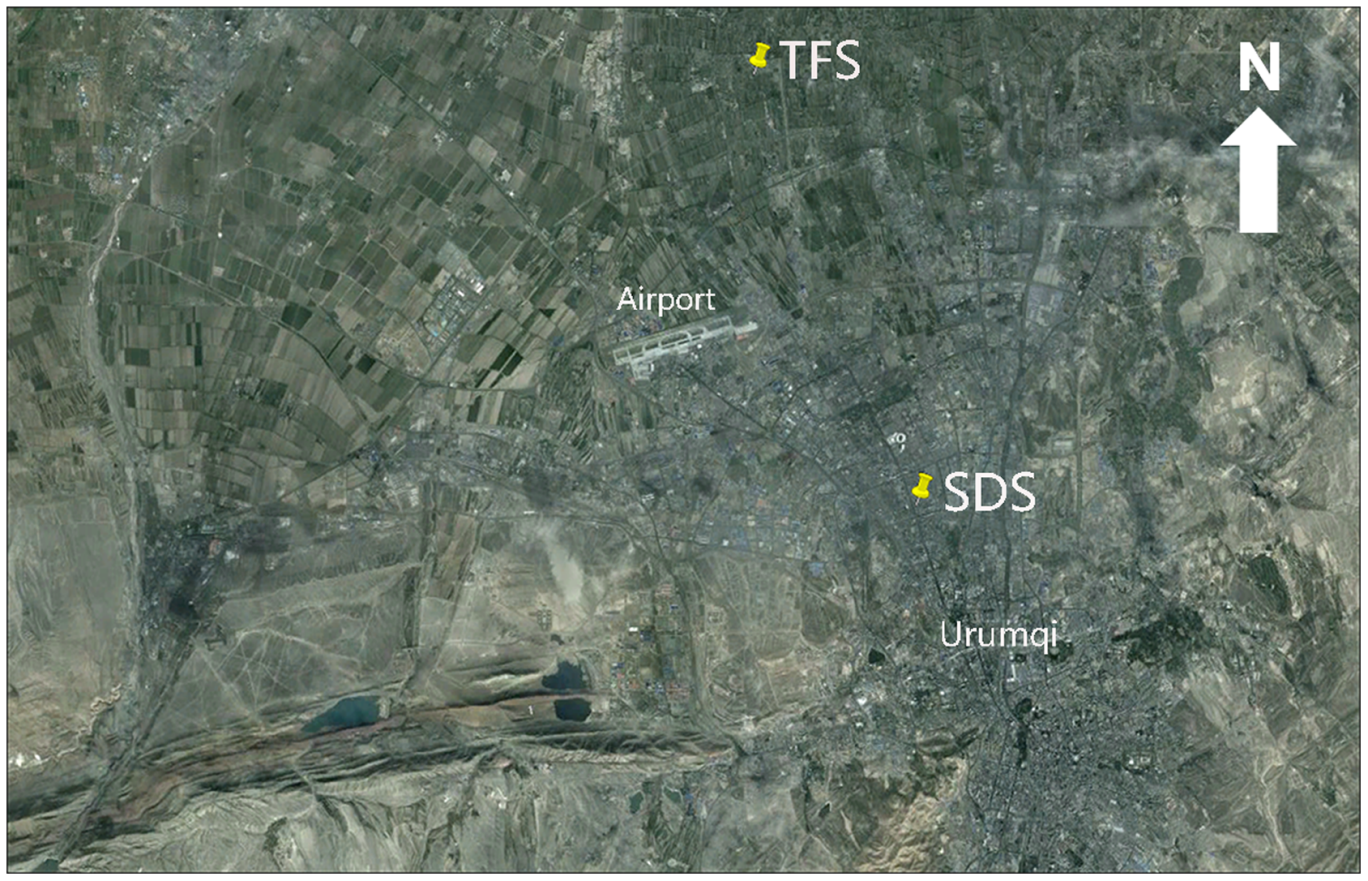
Distribution of two monitoring sites in Urumqi, central Asia.

**Figure 2:**
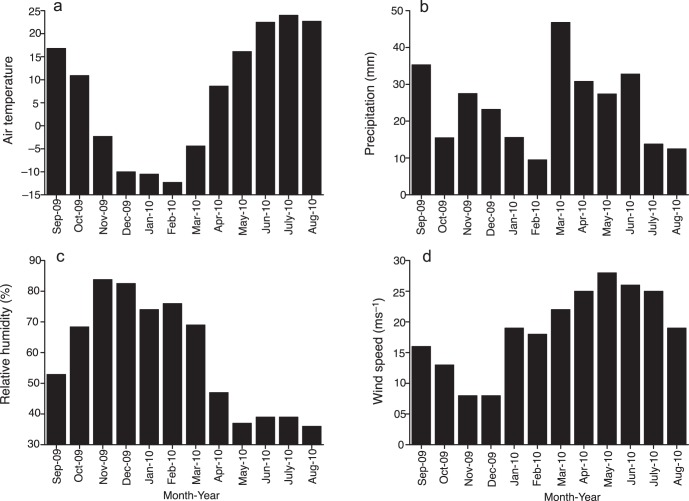
Air temperature, precipitation, wind speed and relative humidity from September 2009 to August 2010 in Urumqi.

Urumqi covers a total area of approximately 140 km^2^ and is one of the most rapidly growing large cities in China whose permanent residents increased from 1.3 million (1990) to more than 2.5 million by 2008 [13]. The study was conducted at two sites, Shengdisuo (SDS) and Tufeisuo (TFS) (Fig. 1). Monthly mean air temperature, precipitation, wind speed and relative humidity from September 2009 to August 2010 in Urumqi are shown in Fig. 2, data provided by China Meteorological Administration at http://cdc.cma.gov.cn. The site at SDS (43°51′ N, 87°34′ E, 775 m a.s.l.) is located at Xinjiang Institute of Ecology and Geography, Chinese Academy of Sciences, and is an urban monitoring site which may be polluted by emissions from motor vehicles and home heating in winter. The site at TFS (43°56′ N, 87°28′ E, 576 m a.s.l.) is located at an experimental farm of Xinjiang Academy of Agricultural Sciences and is a suburban monitoring site which may be polluted by emissions from ammonia in agricultural fields and the use of coal for heating in winter. The distance between the two sites is about 11 km.

### Sampling Procedure and sample Analysis

#### 1. NH_3_ and NO_2_

NH_3_ and NO_2_ samples were collected using Radiello® passive samplers (Aquaria Italy, Trident Equipments Pvt. Ltd., Mumbai, India). Three NH_3_ and three NO_2_ samplers per site at each period were exposed in a PVC shelter (2 m above the ground) which protected the samplers from precipitation and direct sunlight. The NH_3_ and NO_2_ concentrations at both sites were measured monthly by exposing the samplers for two weeks in the middle of each month. After sampling, absorption cartridges of the passive samplers were placed in airtight plastic tubes and stored in a refrigerator at 4°C until analysis with an automated segmented flow continuous flow analyzer (Seal AA3, Norderstedt, Germany) within two months. Detailed information about the passive samplers has been provided by Shen et al. [17]. NH_3_ data were collected from September 2009 to August 2010 and NO_2_ data were collected from February 2010 to December 2011 at both sampling sites (there was no sampling in January 2011 at the TFS site).

#### 2. HNO_3_

HNO_3_ vapour was measured with an active DELTA (DEnuder for Long Term Atmospheric sampling) system designed by the Centre for Ecology and Hydrology, Edinburgh, UK. Samplers for HNO_3_ were coated with a solution of 1% (m/v) K_2_CO_3_ and 1% (m/v) glycerol in methanol and were 15 cm in length. The sampling period was usually one month with a sampling rate of 0.35 L min^−1^. After exposure, the denuders were extracted in 10 mL 0.05% H_2_O_2_ solution and analyzed for NO_3_^−^ using ion chromatography (DX-120, Dionex, Sunnyvale, CA). Because of the lack of suitable instruments, HNO_3_ (vapor) was measured only at TFS from November 2010 to October 2011.

#### 3. PM_10_, pNH_4_^+^ and pNO_3_^−^

Airborne PM_10_ particles were sampled using a particulate sampler (BGI, Omni, Waltham, MA) with a flow rate of 5 L min^−1^ and 7–10 daily samples of PM_10_ were collected per month at each site. The sampler was placed about 2 m above the ground and ran for 24 h to obtain a particulate matter sample on 47 mm quartz filters (Whatman, Maidstone, UK). Before and after sampling, each filter was conditioned for at least 24 h inside a chamber at a relative humidity of 50% and a temperature of 25°C and then weighed (Sartorius, Göttingen, Germany; precision: 1 µg). PM_10_ mass concentrations were determined from the mass difference and the sampled air volume. Each sampling filter was extracted with 10 ml deionized water by ultrasonication for 30 min and the extract solution was filtered through a syringe filter (0.45 mm, Tengda Inc., Tianjin, China) and stored in a refrigerator. Chemical analysis of PM_10_ was conducted within two months. Ammonium and nitrate in PM_10_ (pNH_4_^+^ and pNO_3_^−^) were measured by continuous flow analyzer (Seal AA3). PM_10_ (atmospheric particulate matter having an aerodynamic diameter smaller than 10 µm, which is an air-suspended mixture of solid and liquid particles that vary in size, shape, and chemical composition), NH_4_^+^ and NO_3_^−^ concentrations in PM_10_ (pNH_4_^+^and pNO_3_^−^) were collected from September 2009 to August 2010 at both sampling sites.

#### 4. NH_4_-N and NO_3_-N in precipitation

Precipitation samples were collected daily with stainless steel buckets (SDM6A, Tianjin Weather Equipment Inc., China) at both sites and stored in clean plastic bottles immediately after each precipitation event, then immediately placed in a freezer until analysis of NH_4_-N and NO_3_-N by continuous flow analyzer (Seal AA3) within two months. The stainless steel buckets were cleaned with deionized water before rain/snow collection to avoid contamination and dry deposition. Wet deposition fluxes of NH_4_-N and NO_3_-N were calculated using the N concentrations and the amount of precipitation for each event [18].

QA (Quality Assurance) and QC (Quality Control) are strictly guaranteed in our laboratory as part of the Key Laboratory of Biogeography and Bioresource in Arid Land, Xinjiang Institute of Ecology and Geography, Chinese Academy of Sciences. We used both standard samples and blank samples to control both QA and QC in our study.

### Statistical Analysis

Values of NH_3_, NO_2_, PM_10_, pNH_4_^+^ and pNO_3_^−^ concentrations each month at both sites are means ± standard errors (s.e.). Spearman rank correlation analysis at a significance of 99% between N species and air temperature, precipitation, wind speed and relative humidity was calculated. Differences in Nr species between the two sites were assessed by two–independent–samples test (if *p*<0.05 or 0.01). All statistical analysis was performed using SPSS 11.0 (SPSS, Inc. 2001). Dry deposition fluxes of NH_3_, NO_2_, pNH_4_^+^, pNO_3_^−^ and HNO_3_ were then calculated using the N concentrations and their deposition velocities taken from the literature [19] and these deposition velocities of Nr species being average values in different ecosystem types in China. Wet deposition fluxes of NH_4_-N and NO_3_-N were calculated using the N concentrations and the amount of precipitation for each event by the following equations: N deposition per event (g N ha^−1^) = precipitation (mm)×NH_4_-N or NO_3_- (N concentration in precipitation) (mg N L^−1^)×10, N deposition per month (kg N ha^−1^) = ∑0.001×N deposition per event in a month [18]. All abbreviations for notations are summarized in Table 1.

**Table 1:** A list of abbreviation for notations.

Abbreviation	Explanation
PM_10_	Atmospheric particulate matter having an aerodynamic diameter smaller than 10 µm, which is an air-suspended mixture of solid and liquid particles that vary in size, shape, and chemical composition.
pNH_4_^+^	NH_4_^+^ concentration in PM_10_
pNO_3_^−^	NO_3_^−^ concentration in PM_10_
Nr	Reactive nitrogen
DELTA	DEnuder for Long Term Atmospheric sampling
SDS	ShengDiSuo, an urban sampling site in Urumqi
TFS	TuFeiSuo, a suburban sampling site in Urumqi

## Results

### Concentrations of Nr Compounds in Air

#### 1. NH_3_ concentrations

**Figure 3:**
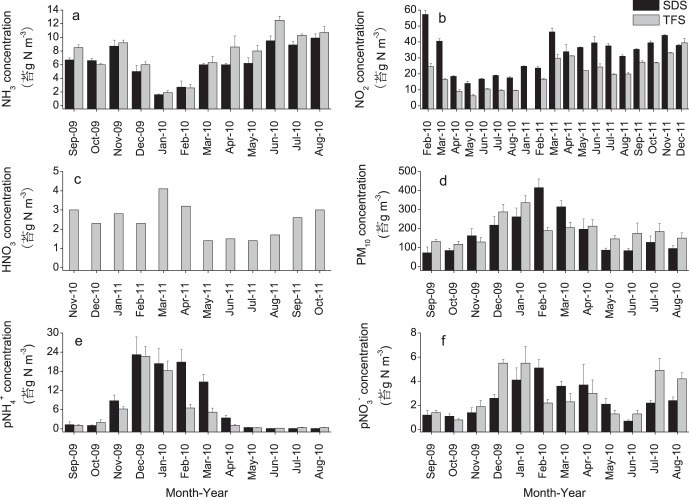
Monthly mean concentrations of (a) NH_3_, (b) NO_2_, (c) HNO_3_, (d) PM_10_, (e) NH_4_^+^ in PM_10_ and (f) NO_3_^−^ in PM_10_ at SDS and TFS from September 2009 to August 2010.

NH_3_ exhibited distinct and significant temporal variation with lower concentrations in winter time (from December to March) than from April to October at both sites (Fig. 3a). Monthly mean NH_3_ concentrations varied within the range 1.6 to 9.9 µg N m^−3^ at SDS and 1.9 to 12.5 µg N m^−3^ at TFS. The annual average NH_3_ concentration was 6.5 µg N m^−3^ at SDS and lower than TFS (7.6 µg N m^−3^). The comparison of monthly mean concentrations between sites indicates no significant difference (*p*>0.05, two–independent–samples tests). NH_3_ concentration showed significant positive correlations with air temperature at SDS and TFS (r = 0. 832 and 0.879, both *p*<0.01) and negative correlations with relative humidity at TFS (*r* = −0.833, *p*<0.01).

#### 2. NO_2_ concentrations

Monthly mean NO_2_ concentrations ranged from 14.0 to 57.3 µg N m^−3 ^at SDS and from 6.2 to 39.5 µg N m^−3 ^at TFS (Fig. 3b). Two–independent–samples tests indicate that NO_2_ concentrations at SDS were significantly higher than at TFS (*p*<0.01). The annual average NO_2_ concentration was 32.3 µg N m^−3^ at SDS, which was higher than at TFS (20.9 µg N m^–3^). NO_2_ concentration showed no significant correlations with air temperature, relative humidity, wind speed or precipitation at either site (both *p*>0.05).

#### 3. HNO_3_ concentrations

Monthly mean concentrations measured for HNO_3_ are given in Fig. 3C. The values range from 1.4 to 4.1 µg N m^−3 ^at TFS. The average concentration was 2.4 µg N m^−3^. The HNO_3_ concentrations show lower values in summer than in winter, autumn or spring.

#### 4. PM_10_ concentrations

Monthly mean PM_10_ concentrations ranged from 72.6 to 415.4 µg m^−3^ at SDS and from 117.6 to 335.8 µg m^−3^ at TFS (Fig. 3d). The comparison of monthly mean concentrations between sites indicates no significant difference (*p*>0.05). PM_10_ concentrations reached their highest values in February (415.4 µg m^−3^) at SDS and in January (335.8 µg m^−3^) at TFS, and the concentrations showed lower values during summer and autumn at both sites. The annual average PM_10_ concentrations were 176.9 and 188.8 µg m^−3^ at SDS and TFS. PM_10_ concentrations were strongly or weakly correlated to air temperature (*r* = −0.831, *p*<0.01 and −0.589, *p*<0.05), pNH_4_^+^ concentrations (*r* = 0.754 and 0.709, both *p*<0.01) and pNO_3_^−^ concentrations (*r* = 0.895 and 0.782, both *p*<0.01) at SDS and TFS, respectively.

#### 5. pNH_4_^+^ concentrations

Monthly mean concentrations of pNH_4_^+^ ranged from 0.1 to 23.2 µg N m^−3^ and from 0.2 to 22.7 µg N m^−3^ at SDS and TFS, respectively (Fig. 3e). The comparison of monthly mean concentrations between sites indicates no significant difference (*p*>0.05). The annual average pNH_4_^+^ concentrations were 7.9 and 5.3 µg N m^−3 ^at SDS and TFS, respectively. The pNH_4_^+^ concentrations indicate that particulate ammonium concentrations peaked during winter months (20.4–23.2 µg N m^−3^) at SDS and reached minimum values in summer (0.2–0.4 µg N m^−3^) at TFS. The pNH_4_^+^ concentrations were correlated to air temperature (*r* = −0.951 and −0.870) and relative humidity (*r = *0.868 and 0.905) at SDS and TFS, respectively.

#### 6. pNO_3_^−^ concentrations

Monthly mean concentrations of pNO_3_^−^ ranged from 0.7 to 5.1 µg N m^−3^ at SDS and 0.8 to 5.5 µg N m^−3^ at TFS (Fig. 3f). The comparison of monthly mean concentrations between sites indicates no significant difference (*p*>0.05). The annual average pNO_3_^−^ concentrations were 2.5 and 2.8 µg N m^−3 ^at SDS and TFS, respectively. The pNO_3_^−^ concentrations indicate that particulate nitrate concentrations reached their highest values in February (5.1 µg N m^−3^) at SDS and in December and January (5.5 µg N m^−3^) at TFS.

### Concentrations of Inorganic N Species in Precipitation

#### 1. NH_4_– N concentrations

**Figure 4:**
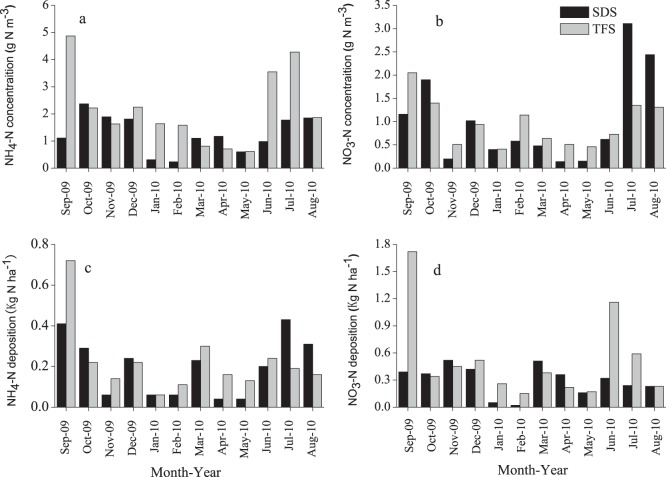
Monthly mean concentrations of (a) NH_4_-N, (b) NO_3_-N in precipitation and wet deposition fluxes of (c) NH_4_-N and (d) NO_3_-N at SDS and TFS from September 2009 to August 2010.

Monthly mean concentrations of NH_4_–N in precipitation ranged from 0.2 to 2.4 mg N L^−1^ at SDS and from 0.6 to 4.9 mg N L^−1^ at TFS (Fig. 4a). Annual mean NH_4_– N concentrations were 1.3 and 2.2 mg N L^−1^ at SDS and TFS, respectively. Paired–samples t-test (95% confidence interval) indicates that the NH_4_–N concentration at SDS was significantly lower than that at TFS.

#### 2. NO_3_– N concentrations

Monthly mean concentrations of NO_3_– N in precipitation ranged from 0.1 to 3.1 mg L^−3^ at SDS and from 0.4 to 2.1 mg L^−3^ at TFS (Fig. 4b). The monthly mean NO_3_– N concentrations at both sites showed no significant difference (*p*>0.05, paired samples t-test). The annual average NO_3_– N concentration was 1.0 mg N L^−1^ at both SDS and TFS. The higher NO_3_–N concentrations measured at SDS and TFS occurred mostly in summer and autumn.

### Dry and Wet Deposition of Inorganic N

Dry deposition fluxes of Nr species NH_3_, NO_2_, HNO_3_, pNH_4_^+^ and pNO_3_^−^ were estimated by the measured Nr concentrations and their deposition velocities taken from the literature (Table 2). The deposition fluxes of NH_3_, NO_2_, pNH_4_^+^ and pNO_3_^−^ averaged 6.2, 5.9, 9.2 and 3.7 kg N ha^−1^ yr^−1^, respectively, across the two sites. The deposition flux of HNO_3_ was 13.4 kg N ha^−1^ yr^−1 ^at TFS. Monthly mean wet deposition values of NH_4_-N and NO_3_-N at both sites are shown (Fig. 4c, 4d). Annual wet deposition fluxes of NH_4_-N were 3.6 and 6.2 kg N ha^−1^ yr^−1^ and those of NO_3_-N were 2.4 and 2.6 kg N ha^−1^ yr^−1 ^at SDS and TFS, respectively. Mean inorganic N wet deposition was 7.4 kg N ha^−1^ yr^−1^ across the two sites. Because there was no measurement of HNO_3_ at SDS, as a first approximation we assume the HNO_3_ concentration to be the same at both sites. Total dry and wet N deposition rates were up to 46.7 and 44.8 kg N ha^−1^ yr^−1^ at the urban (SDS) and suburban (TFS) sites.

**Table 2:** Dry and wet N deposition fluxes at SDS and TFS from September 2009 to August 2010.

N_r_	Concentration^b^		V_d_ d		Deposition	
species^a^	Mean±s.e.^c^ (µg N m^−3^/g N m^−3^)		(cm s^−1^)		Mean±s.e. (kg N ha^−1 ^yr^−1^)	
	SDS	TFS	SDS	TFS	SDS	TFS
NH_3_	6.5±0.5	7.6±0.6	0.28	0.28	5.7±0.4	6.7±0.5
NO_2_	32.3±1.3	20.9±0.9	0.07	0.07	7.1±0.3	4.6±0.2
pNH_4_^+^	7.9±1.7	5.3±0.9	0.44	0.44	11.0±2.4	7.4±1.2
pNO_3_^−^	2.5±0.5	2.8±0.6	0.44	0.44	3.5±0.7	3.9±0.8
HNO_3_	2.4±0.2	2.4±0.2	1.77	1.77	13.4±1.1	13.4±1.1
Sum(N_d_ a)	–	–	–	–	40.7	36.0
NH_4_-N	1.3	2.2			3.6	6.2
NO_3_-N	1.0	1.0			2.4	2.6
Sum(N_w_ a)	–	–	–	–	6.0	8.8

a N_d_ and N_w_ denote N dry and wet deposition, respectively; full names of other Nr species are listed in Table 1;

b “µg N m^−3″^ denotes the unit of atmospheric concentration for NH_3_, NO_2_, pHN_4_^+^, pNO_3_^−^ and HNO_3_ and “g N m^−3^” denotes the concentration unit for NH_4_-N and NO_3_-N in precipitation;

c s.e. denotes standard deviation of means;

d Taken from Zhang et al. (2004). Due to the lack of measurements of HNO_3_ at SDS in this study, as a first estimation we assume the same HNO_3_ concentration at SDS as at TFS.

## Discussion

### Seasonal Variation in Atmospheric Nr Concentrations

The NH_3_ concentration was lower in winter than other times of year at both sites and this is consistent with results reported from the North China Plain [20]. This is most likely due to high NH_3_ emissions from N fertilizer application, rapidly increaing livestock production, and motor vehicles. In north and northwest China NH_3_ concentrations are relatively high in spring and summer mainly due to N fertilization and higher air temperatures, both of which promote NH_3_ volatilization from arable soils. For example, the N fertilizer application rate is up to 240****kg N ha^−1^ per crop to achieve high yields in spring and summer at suburban parts of Urumqi. However, less than 30% of applied N fertilizer was taken up by the crops [21] and much of the unaccounted-for fertilizer N was lost by NH_3_ emissions. This may be an important reason for high NH_3_ concentration in rural or suburban area. Source strength and removal efficiency can explain seasonal variation in NH_3_ concentrations [22]. During warm months NH_3_ emissions will be promoted from other sources such as animal stables, domestic toilets and fertilized soils. Therefore, NH_3_ concentrations were higher at TFS (suburban) than SDS (urban) in summer. Because of zero fertilizer application and the very low air temperatures in winter, NH_3_ concentrations were low at both sites. Moreover, the contribution of motor vehicles to NH_3_ emissions is negligible in suburban areas where agriculture is the main source of NH_3_, while motor vehicles can be main sources of ammonia in the urban environment [23]. In the current study NO_2_ concentrations exhibited distinct and significant temporal variation, with higher concentrations between February and March at both sites. These NO_2_ values were very similar to the high concentrations observed in other regions of China [17], [24]. The area of Urumqi is only 140 km^2^ with a population of 2.2 million and the total number of motor vehicles in the city was 140,000 units in 2003. However, motor vehicles had reached 240,000 units and were increasing at a rate of 260 units per day since 2009. Vehicular emissions contribute significantly to air contamination in urban areas and heavy-duty vehicles are the main contributors to particle and NO_x_ emissions [25]. For example, NO_x_ emissions in the urban area (SDS) were mainly from heavy duty passenger buses, which comprised approximately 41.3% of total vehicle numbers, including passenger cars, buses, motorcycles, and trucks [26]. Therefore the higher NO_2_ concentrations are likely related to anthropogenic sources such as vehicle emissions at the urban site. In addition, the use of coal for heating in Urumqi city in winter could be the main explanation for the increased NO_x_ concentration in the atmosphere. Other factors such as the dry weather, temperature inversion and low wind speed in winter will also lead to the accumulation of atmospheric NO_x_ because these conditions do not favor the transformation and diffusion of atmospheric pollutants.

HNO_3_ concentrations at the suburban site (TFS) in our study were much higher than at many other sites across the world. For example, the HNO_3_ concentration was 0.6 µg N m^−3^on the North China Plain [17], 0.34 µg N m^−3 ^on an urban-facing site on Mt. Gokurakuji 0.09 µg N m^−3^ in Kobe city in Japan [27], [28], 0.59 µg N m^−3^at Montelibretti in Italy and 0.60 µg N m^−3^at Paterna in Spain [29]. Urumqi has some of the highest air pollution in China due to the large amounts of coal combustion for industrial development and residential heating resulting in high HNO_3_ concentrations. The high concentrations of NO_2_, NH_3_ and HNO_3_ species indicate substantial atmospheric Nr pollution in Urumqi.

The PM_10_ concentrations at SDS and TFS were much higher than reported from numerous other urban and suburban sites [30]–[32] but lower than the values from Lanzhou, a city with some of the highest air pollution worldwide with a peak PM_10_ concentration (541.9 µg m^−3^) occurring in April due to dust events [33] and Tazhong area in the Taklimakan Desert [34]. The highest PM_10_ concentrations occurred in winter due to domestic heating in Urumqi. In addition, fireworks are a traditional way to celebrate the Chinese New Year (Spring Festival, in January or February) and may increase some pollutant concentrations in aerosols during the Spring Festival period [35]. Moreover, increasing numbers of vehicles, low wind speed and high humidity in winter (Fig. 2) have resulted in considerable PM_10_ pollution in Urumqi. For example, vehicle emissions have become a major source of PM in Urumqi due to a rapid growth in vehicle population over the past ten years. PM emissions in the urban area (SDS) were mainly from heavy duty trucks, which comprised approximately 46.6% of total of vehicle numbers, gasoline and diesel engine vehicles and traffic density also contributed to particle and NO_x_ emissions, gasoline engine vehicles comprising approximately 64% of total vehicle numbers in Urumqi [26], and diesel vehicles in urban areas across Europe are equipped with exhaust after-treatment systems which reduce the total mass of emitted particles [36]. Therefore, to protect human health in urban regions we should control vehicle emissions as the highest priority [37]. The pNH_4_^+^ concentrations at the both sites were much higher than those at many other urban and suburban sites [38]–[40]. This was most likely generated from coal combustion in winter and from other anthropogenic sources such as vehicles, manufacturing and industrial processes at other times of year. Although wind speed varied slightly between seasons, other meteorological parameters such as low temperature and high relative humidity point towards poor dilution of pollutants during the winter period. Regarding the presence of nitrate associated with ammonium, it was more likely to form secondary particulate ammonium nitrate during the cold and humid periods in Urumqi. The pNO_3_^−^ concentration was correlated to the pNH_4_^+^ concentration at the urban site (*r* = 0.680, *p*<0.05). Moreover, most anions and cations in PM_10_ originate from coal combustion in winter, indicating increasingly anthropogenic induced aerosols to be concentrated in Urumqi [16].

Such high Nr dry concentrations (39.0–51.6 µg N m^−3^) at SDS and TFS reflect serious air pollution due to increased emissions by traffic, industry and domestic heating which affect the eutrophication of water bodies and soil acidification in neighboring natural and semi-natural ecosystems, but the Nr compounds may make a substantial contribution to nutrient budgets in some intensively managed croplands [41] or in some low input agroecosystems. N inputs from wet and dry deposition are estimated at 15 Tg N yr^−1^ for the whole of China in the 2000s and about 5.0 Tg annual N deposition may have returned to croplands in China since the 2000s [15]. This is an important nutrient input to croplands from the atmosphere.

### Inorganic N Concentrations in Precipitation

It is generally thought that the major anthropogenic source of NH_4_^+^ in precipitation is NH_3_ volatilized from N fertilizers and the excrement of human beings and animals, major anthropogenic sources of NO_3_^−^ are NO_x_ emitted from fossil fuel combustion from power plants, automobiles, and biomass burning [42]. In the present study the higher NH_4_-N concentrations at the suburban site (TFS) and higher NO_3_-N concentrationsat the urban site (SDS) were found in summer and early autumn (Fig. 4), reflecting the effects of both agricultural and non-agricultural (e.g. traffic and industrial) Nr sources. In recent years motor vehicles have become important sources of ammonia from cars and other vehicles with catalytic converters emitting large amounts of NH_3_ [43] and this might increase NH_4_^+^ concentrations in precipitation as NH_3_ can transform to pNH_4_^+^. We found significant positive correlations between NO_3_^–^ and NH_4_^+^ in precipitation at SDS (*r* = 0.583, *p*<0.05) and TFS (*r* = 0.736, *p*<0.01), suggesting similar origins for a substantial component of the ammonium- and nitrate-N. The ratio of NH_4_-N to NO_3_-N (expressed as NH_4_-N/NO_3_-N) was calculated to analyze the source information on the wet deposition in this study area. In general, the ratio of NH_4_-N to NO_3_-N reflects the relative contribution of Nr from industry and transportation, agriculture, and animal husbandry to N deposition on a local scale [44], NH_4_-N/NO_3_-N is usually much smaller than 1 in industrialized regions and the ratio is usually greater than 1 in intensive agricultural regions [45]. We found annual average NH_4_-N/NO_3_-N values of 1.3 and 2.2 at SDS and TFS and this indicates that NH_4_-N concentrations in precipitation are likely be largely influenced by agriculture and human and animal excrement, NH_3_ may remain the major contributor to wet N deposition in our study area, especially at the suburban site, compared with the NO_3_-N mainly from fossil fuel combustion in industry and transportation [46].

### Fluxes of Dry and Wet Deposition of Inorganic N

The mean NH_3_ deposition flux in Urumqi was 6.2 kg N ha^−1^ yr^−1^, which was lower than the mean value (18.4 kg N ha^−1^ yr^−1^) on the North China Plain [20]. Mean NO_2_ deposition flux in Urumqi averaged 5.9 kg N ha^−1^ yr^−1^, which is slightly higher than the average value (3.03 kg N ha^−1^ yr^−1^) for China [47] but much lower than the reported value (17.3–30.7 kg N ha^−1^ yr^−1^) on the North China Plain [17]. The total particle N deposition fluxes (pNH_4_^+^ and pNO_3_^−^) were 14.4 and 11.2 kg N ha^–1^ yr^−1 ^at SDS and TFS. Our results are similar to those on the North China Plain (8.4–14.2 kg N ha^−1^ yr^−1^) [17] and a suburban region of Gpoalpura in India reported by Satsangi et al. [48] but higher than in Thessaloniki city in Greece (0.99 kg N ha^−1^ yr^−1^) [49] or Al-Hashimya city in Jordan (0.76 kg N ha^−1^ yr^−1^) [50].

In the present study the wet deposition fluxes (7.4 kg N ha^−1^ yr^−1^ on average) were relatively low compared to those reported for other areas in China. For example, wet deposition fluxes of inorganic N were 24–27 kg N ha^−1^ yr^−1 ^in east China [51] and 27–30 kg N ha^−1^ yr^−1 ^on the North China Plain [18], [52]. Lu and Tian [47] reported an average value of 9.9 kg N ha^−1^ yr^−1^ in China. The relatively low wet deposition of inorganic N in Urumqi may have been largely due to the low precipitation in this arid region, with mean annual precipitation of about 259 mm from 1960 to 2004 [53]. Ratios of NH_4_-N to NO_3_-N in precipitation (1.3 and 2.2 at SDS and TFS, respectively) clearly show the impacts of agricultural (suburban site) and non-agricultural (urban site) sources on wet deposition of N, and further reflects the effects of NOx emissions from industry and transportation in urban areas (Pan et al., 2012) [46]. Moreover, dissolved organic N comprises approximately 30% of total N deposition [54], but organic N in wet deposition was not considered in this study. To obtain more accurate estimates of the wet N deposition in this study area it will be necessary to analyze the organic N concentrations., The total N depostion (dry and wet) fluxes were systematically evaluated in this study. Whereas previous studies only focused on the wet deposition (Xu et al., 2008) [16] and the dry deposition of HNO_3_ and Nr species were not measured. Total N deposition (dry and wet) fluxes were 46.7 and 44.8 kg N ha^−1^ yr^−1^ at the urban and suburban sites in Urumqi, with a mean value of 45.8 kg N ha^−1^ yr^−1^. Such deposition rates are somewhat higher than the critical load of N for sensitive ecosystems (e.g., 5–10 kg N ha^−1^ yr^−1^) according to Bobbink and Roelofs [55]. We found the proportion of Nr dry deposition in total deposition to be up to 80.6%, much higher than in other humid regions worldwide [56]. Nr dry deposition is a very sensitive index for croplands, grasslands, forests and aquatic ecosystems and it reflects atmospheric pollution levels, while processes that control N deposition velocities include gravitational settling, impaction, and diffusion, processes which act simultaneously and are affected by many variables including particle size, wind speed, relative humidity, and land surface roughness [57]. Because of the difficulty in obtaining in-situ dry deposition velocities of Nr species [58], we used simplified deposition velocities taken from the literature [19]. We are therefore aware of the uncertainty of dry deposition of inorganic N in the current study. To reduce uncertainties of N deposition, wet and dry deposition of organic N and the in-situ measurements of N dry deposition velocities merit evaluation in an arid region of central Asia in future studies. In addition, modeling tools are very useful for quantifying atmospheric N deposition (including both spatial and temporal variations), but model validation generally suffers from a serious lack of long-term Nr concentrations monitoring data. Therefore, modeling tools and long-term monitoring will be conducted in future studies in this region.

## Conclusions and Summary

1. Annual mean concentrations of NH_3_, NO_2_, HNO_3_, pNH_4_^+^, pNO_3_^−^, and PM_10_ were 7.1, 26.6, 2.4, 6.6, 2.7 µg N m^−3^ and 182.9 µg m^−3^ across both urban (SDS) and suburban (TFS) sites in Urumqi, central Asia. The NO_2_, pNH_4_^+^ and pNO_3_^−^ concentrations were highest during winter and the peak NH_3_ concentrations appeared in summer, reflecting the distinct impacts of agricultural (NH_3_) and non-agricultural (other Nr species) sources. Such high Nr concentrations imply serious local air pollution due to increased emissions from traffic, industry and domestic heating in winter.
2. The annual mean NH_4_-N and NO_3_-N concentrations in precipitation were 1.8 and 1.0 mg L^−1^, respectively, across SDS and TFS. The higher NO_3_-N and NH_4_-N concentrations occurred mostly in summer and early autumn, and significant positive correlations were found between NO_3_-N and NH_4_-N. The annual mean NH_4_-N/NO_3_-N values were 1.3 and 2.2 at SDS and TFS, respectively.
3. Dry deposition fluxes of NH_3_, NO_2_, HNO_3_, pNH_4_^+^ and pNO_3_^−^ averaged 6.2, 5.9, 13.4, 9.2 and 3.7 kg N ha^−1^ yr^−1 ^at the two sites with a total dry deposition flux of 38.4 kg N ha^−1^ yr^−1^. Wet deposition fluxes of NH_4_-N and NO_3_-N were 4.9 and 2.5 kg N ha^−1^ yr^−1^ across both suburban and urban sites in central Asia. The total (dry and wet) N deposition values averaged 45.8 kg N ha^−1^ yr^−1^, with dry deposition accounting for a high percentage (83.8%) of total deposition in Urumqi in an arid environment. Such high N deposition implies that heavy environmental pollution needs to be controlled by substantially reducing the Nr emissions to the environment, but high Nr dry deposition can be used as an important agricultural nutrient resource and it should be considered in integrated N fertilizer management in arid regions.
