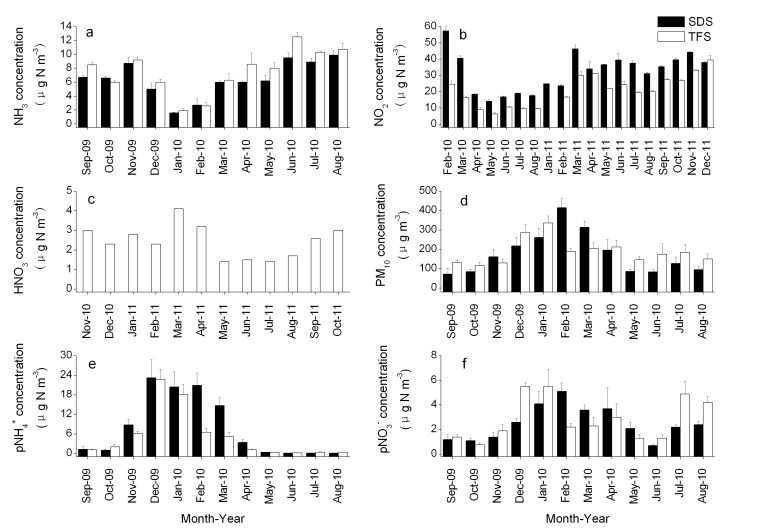# Correction: Atmospheric Nitrogen Deposition at Two Sites in an Arid Environment of Central Asia

**DOI:** 10.1371/annotation/9f5d3b10-1021-4d7d-8bad-26e9b513176b

**Published:** 2014-01-10

**Authors:** Kaihui Li, Xuejun Liu, Wei Song, Yunhua Chang, Yukun Hu, Changyan Tian

In Figure 2A, the units for Air Temperature should be ℃. In Figure 3 all of the 苔 characters should be μ. 

Please see the corrected Figure 2 here: 

**Figure:**
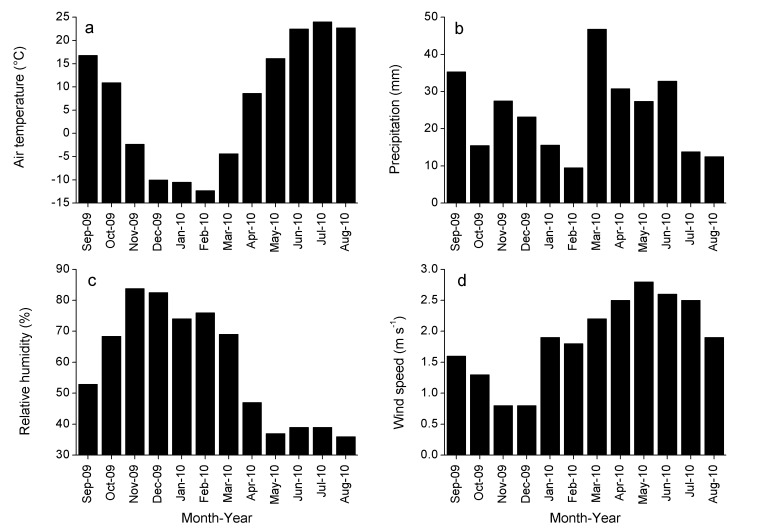


Please see the corrected Figure 3 here: 

**Figure:**